# Combination of Novel Therapies for HDV

**DOI:** 10.3390/v14020268

**Published:** 2022-01-28

**Authors:** Menashe Elazar, Jeffrey S. Glenn

**Affiliations:** 1 Division of Gastroenterology and Hepatology, Department of Medicine-Microbiology and Immunology, Stanford University School of Medicine, Stanford, CA 94305, USA; menashe@stanford.edu; 2 Palo Alto Veterans Administration, Palo Alto, CA 94305, USA

**Keywords:** HDV, lonafarnib, peginterferon lambda

## Abstract

Treatment options for HDV have been limited to interferon alfa-based therapies with its poor efficacy to side effects ratio. Several novel therapies have now advanced into the clinic. As they each have a different mechanism of action, there is the potential for combination therapy. Here we review how studying the HDV life cycle has led to the development of these novel therapies, the key developments leading to, and the details of, the first combination study of novel anti-HDV therapies, and suggest what additional combinations of novel therapies can be anticipated as we enter this exciting new area of HDV treatments.

## 1. Introduction

Hepatitis Delta virus (HDV) is estimated to afflict between 5–10% of hepatitis B virus (HBV)-infected patients worldwide [1]. While in many developed countries HDV prevalence is within that single digit range, in some countries, the rate is much higher (33% in Mauritania [2], 66% in Gabon [3,4] and between 60–88% in Mongolia [5,6]). In some countries, even though the prevalence of HDV is low, some regions within these countries demonstrate a very high rate of HDV positivity [7]. Thus, the prevalence of HDV is highly variable, and migration from countries in which HDV is endemic may shift the prevalence to higher rates in developed countries [8,9]. Suboptimal testing and the use of unreliable HDV diagnostics makes this landscape even more complicated and may suggest that we are still missing the true HDV infection rate in many countries.

The clinical manifestations of HDV co-infection are not different than those of HBV mono-infection, except that HDV infection generally results in a more deleterious liver disease with a faster progression to cancer and end-stage liver disease [10]. While theoretically, complete eradication of HBV in a patient could eventually lead to resolution of the accompanying HDV, anti-HBV therapies that leave residual amounts of HBsAg and integrated HBV genomes will be insufficient to result in HDV clearance. As such, sterilizing anti-HBV therapies would be required to eradicate HDV, and realistically, these remain out of reach for the foreseeable future. Effective anti-HDV therapies are urgently needed.

No FDA-approved therapy for HDV is available, and for many years, the only potential treatment for HDV infection has been interferon α-2a (IFNα), a minimally effective treatment with a challenging side-effect profile and a high rate of relapse after cessation of treatment [11]. The first indication that a specific anti-HDV therapy may be possible emerged when it was shown that HDV depends on prenylation, a host cell-derived lipid modification of the HDV large antigen, for its assembly and completion of its life cycle [12]. This finding opened a new era for antiviral treatment for HDV infection. Since then, several new anti-HDV therapies that target distinct stages in the HDV life cycle have been discovered and are now in different phases of clinical development.

In this review, we will discuss the HDV life cycle and briefly describe the findings that brought about the development of new anti-HDV agents for the clinic, with an emphasis on the first two novel agents to be used in combination therapy. Combination therapy, consisting of therapies with different, distinct mechanism of actions, will likely be the cornerstone of treatment and cure for HDV.

## 2. HDV Life Cycle and Targets for Antiviral Intervention

HDV is an enveloped virus with a small circular single stranded, negative-sense RNA genome that is always associated with HBV infection. The HDV particle consists of a 1.7 kb RNA genome that is present in a collapsed rod structure and associated with delta antigen, the only protein expressed by the viral genome [13]. Delta antigen has two forms, small and large delta antigen (SHDAg and LHDAg, respectively) [14]. The viral genome and the delta antigen proteins are encapsulated within a lipid envelope that is embedded with the full complement of HBV surface antigen (HBsAg) proteins. As for HBV, this HBsAg mediates HDV entry into its target liver cell [15]. The requirement for HBsAg in entry, assembly and release explains the clinical dependance of HDV infection on co-infection with HBV.

The HBV entry receptor, human sodium-taurocholate co-transporting polypeptide (hNTCP) serves as the entry receptor for HDV [16] through the same mechanism of binding, involving a 48 amino acid stretch in the pre-S1 domain in the N-terminus of the large form of HBsAg, L-HBsAg [17].

Upon entry, the HDV genome is uncoated and translocated to the nucleus where replication occurs in an RNA-dependent manner with no DNA intermediates [15]. The viral genome does not code for a polymerase protein and instead appears to recruit the host DNA-dependent RNA polymerase II [18] for replicating its genome in a rolling circle mechanism with SHDAg serving as a replication co-factor [18].

**Figure 1:**
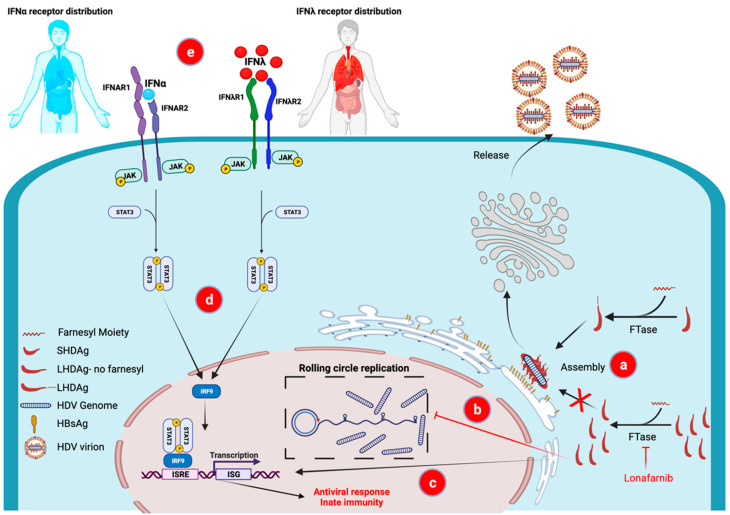
Mechanism of action of the components in the first combination of novel therapies for HDV, lonafarnib and interferon lambda. Inhibition of HDLAg prenylation by the farnesyltransferase inhibitor lonafarnib results in (**a**) virus assembly inhibition and prevention of progeny virus production, (**b**) accumulation of LHDAg in the cells leading to a transdominant inhibition of HDV rolling circle replication and (**c**) induction of innate immunity by activating interferon sensitive gene (ISG) expression. Type III IFNλ results in comparable innate immune antiviral actions as classical Type I IFNα (both activate the same pathways-**d**) The receptor distribution for IFNλ, however, is limited to epithelial cells, including hepatocytes (red shading-**e**), resulting in milder side effects compared to IFNα whose receptors are more widely distributed (most cells in the body including immune cells) (blue shading-**e**) FTase—farnesyltransferase; HBsAg—HBV surface antigen; ISG—interferon stimulated gene; ISRE—interferon sensitive response element; LHDAg—large HDV Antigen; SHDAg—small HDV antigen. Created with BioRender.com.

The HDV genome codes for a single protein, the HDV antigen. This protein exists in two forms, SHDAg and LHDAg, where the large antigen has an extra 19 amino acids added to its C-terminus. The addition of these 19 amino acids to the coding sequence of the SHDAg is a result of an RNA editing event mediated by the enzyme Adenosine Deaminases Acting on RNA-1 (ADAR1), that modifies an amber stop codon on the HDV antigenomic RNA. This modification results in translation proceeding to the next downstream stop codon resulting in the additional 19 amino acids unique to the LHDAg [19]. While SHDAg serves as a replication co-factor, LHDAg serves as transdominant inhibitor of replication [20]. Moreover, LHDAg is essential for particle assembly [21], thus the RNA editing event serves as a molecular switch that enables the shift from replication of the viral genome to assembly and release of infectious virions. The role of LHDAg in virus assembly stems from a CXXX-box motif (where C = cysteine, and X = one of the last 3 amino acids at the carboxy terminus) contained in the last 4 of the 19 amino acids extension of the protein. This motif is a substrate for a farnesylation reaction where the enzyme farnesyltransferase (FTase) adds a farnesyl prenyl lipid moiety to the cysteine of the CXXX-box motif (Figure 1a) [12]. This post translational modification is essential for interaction of the HDV nucleocapsid with the membrane anchored HBsAg to assemble the virus particle [22]. The final stage of the life cycle requires release of the viral particle through the trans-Golgi network where the HDV particle is enveloped with a lipid membrane embedded with the HBsAg and secreted out of the infected hepatocyte [23].

## 3. Translating Molecular Virology into Clinical Anti-HDV Therapeutics

Understanding the molecular mechanisms behind some of the key stages in the HDV life cycle enabled the development of the new therapeutics for HDV. As further detailed below, these new therapies that are currently in various stages of clinical evaluation affect HDV in different stages of the life cycle including entry, assembly, and release, as well as immune activation.

### 3.1. Inhibiting Entry—Myrcludex B

The entry receptor for HBV and HDV into the hepatocyte was identified as hNTCP [16]. It was determined that a 48 amino acid stretch at the N-terminus of the L-HBsAg mediates its interaction with hNTCP and facilitates virus entry [17]. This finding was followed by synthesis of a myristoylated peptide mimic composed of 48 amino acids of the L-HBsAg protein’s N-terminus that was shown to competitively inhibit both HBV and HDV binding to hNTCP, and their entry in vitro [24] and in vivo [25]. Clinical trials of this peptide, Myrcludex B (MyrB), are in progress and although sustained clearance of HDV has not been demonstrated, it was estimated that a 3-year course of daily subcutaneous injection of MyrB will be supportive of an approval for a chronic therapy [26]. To date, MyrB, now being developed under the name of bulevirtide, has only been used in combination with older agents such as tenofovir and pegylated (PEG)-IFNα. Week 24 interim data in a phase 3 study has been reported which showed 37% of patients achieved the composite endpoint of 2 log decline in HDV RNA and ALT normalization with bulevirtide monotherapy [27]. Interestingly, no synergy was observed on this composite endpoint when bulevirtide was combined with PEG IFNα in a phase 2b study, with only 30% of patients achieving 2 log decline in HDV RNA and ALT normalization [28]. In the earlier clinical studies with MyrB, when HDV RNA declined during treatment, it rebounded in most patients after the end of MyrB treatment [29,30]. In a recent case report of 3 patients, a daily administration of MyrB for 48 weeks showed a benefit for the treatment of HDV-related compensated cirrhosis in 2 out of the 3 patients [31].

Conditional approval for bulevirtide monotherapy as a chronic, daily subcutaneous injection was recently granted in Europe. Bulevirtide shows promise as a future antiviral cocktail component in treatments for HDV.

### 3.2. Inhibiting HBsAg Secretion—Nucleic Acid Polymers

Nucleic acid polymers (NAPs) are single-stranded phosphorothioate nucleic acid polymers that were shown to possess antiviral activity against several viruses from different orders [32,33,34,35,36,37]. The antiviral activity of NAPs has been attributed to a variety of disparate mechanisms of action including, entry inhibition [37], their amphipathic nature [36] and, most recently, to the interaction of their hydrophobic face with amphipathic stretches within proteins from various pathogens [32,33,34,35]. Interestingly, NAPs were shown to inhibit HBV infection of duck hepatitis both in vitro and in vivo in entry and post-entry stages [38,39]. These results prompted a clinical trial to test NAPs activity in HBV-infected patients with encouraging results [40], which prompted further study to evaluate the ability of NAPs to reduce HDV RNA in patients. In the first and only trial assessing the activity of NAPs, promising drops in HBsAg and HDV RNA were observed in 12 HDV patients following 30 weekly intravenous infusions of NAPs which was augmented with the addition of PEG-IFNα. Detailed results of the study can be found in [41,42].

### 3.3. Inhibiting Assembly—Lonafarnib, a Farnesyltransferase Inhibitor

The CXXX-box motif of LHDAg was shown to undergo prenylation, namely addition of a farnesyl group to cysteine [12]. Inhibition of FTase, the enzyme that mediates farnesylation of LHDAg, has been shown to inhibit HDV particle production in various systems including virus-like particles [43], primary hepatocytes [22] and an in vivo mouse model [44]. These findings strongly suggested that inhibition of FTase may serve as an antiviral strategy for HDV therapy in the clinic. In addition, in vitro studies have indicated that FTase inhibition can result in increased intracellular LHDAg which is a transdominant inhibitor of HDV genome replication (Figure 1b) [20] and induces innate immunity against HDV (Figure 1c) [45].

The farnesyltransferase inhibitor (FTI) lonafarnib is the only oral agent in clinical development for HDV. Importantly, lonafarnib is also part of the first and only combination of two novel anti-HDV agents, which includes peginterferon lambda (PEG-IFNλ). We will thus first review, in more detail, the clinical studies of lonafarnib and PEG-IFNλ when used separately for HDV, followed by their use together in the first of likely many future trials combining novel agents for HDV.

In a series of five phase 2 clinical studies, over 120 HDV patients have been treated with lonafarnib. The first study, a proof-of-concept study conducted at the National Institutes of Health (NIH), examined orally-administered lonafarnib monotherapy twice daily doses (BID) of 100 mg and 200 mg vs. placebo for 4 weeks with a 24-week follow-up [46]. The study demonstrated several key points: (1) HDV RNA declined in a dose-dependent manner with 0.79 log and 1.6 log decline in the 100 mg and 200 mg lonafarnib BID doses, respectively; (2) HDV RNA decline was strongly correlated to lonafarnib serum concentration; (3) sequencing of viral isolates at all time points revealed no evidence of resistance [46]. Although such a result was predicted for a host-targeting antiviral because the targeted locus is not under genetic control of the virus, this represents some of the first empiric data in humans, demonstrating that a host-targeting antiviral can indeed have a high barrier to the development of resistance.

These findings prompted continuation of lonafarnib clinical development in the phase 2 *LOWR-1* study at the University of Ankara. In this study, lonafarnib was first explored as a monotherapy at 100 mg twice daily (TID), 200 mg BID, and 300 mg BID. Data supported lonafarnib 200 mg BID as the maximal tolerated dose (MTD) with expected gastrointestinal (GI) side effects of diarrhea and nausea [47]. In an effort to increase exposures of lonafarnib while balancing GI tolerability, lonafarnib was next boosted with ritonavir (RTV), an inhibitor of CYP3A4 which is the main metabolizing enzyme of lonafarnib, enabling delivery of lower lonafarnib doses to the GI tract while achieving higher post-absorbed drug levels in the liver. Indeed, addition of RTV 100 mg QD to lonafarnib 100 mg BID achieved substantially higher serum concentrations of lonafarnib compared to the highest 300 mg BID lonafarnib monotherapy dose [47]. Moreover, lonafarnib boosted with RTV allowed patients to achieve a greater (−2.4 log vs. −2.0 log) decline in serum HDV RNA at 4 weeks with significantly milder GI side effects [47]. It is noteworthy that the combination arms of lonafarnib with either RTV or PEG-IFNα demonstrated a rapid virological response achieving 3.2 log and 2.96 log HDV RNA decline, respectively, within 8 weeks [47], compared to the historical PEG-IFNα treatment response that required 48 weeks to achieve a similar result [48].

Next, the phase 2 *LOWR-2* trial was designed to determine doses of lonafarnib boosted with RTV, with or without PEG-IFNα, that could yield desired anti-HDV responses and ALT normalization rates that were sufficiently well tolerated to enable longer-term dosing [49]. Fifty-five patients were enrolled in 10 different regimens that comprised 3 main treatment groups: high dose lonafarnib (lonafarnib ≥ 75 mg po BID + ritonavir) (n = 19, 12 weeks); all oral low dose lonafarnib (lonafarnib 25 or 50 mg po BID + ritonavir) (n = 24, 24 weeks) and combination low dose lonafarnib with PEG-IFNα (lonafarnib 25 or 50 mg po BID + ritonavir + PEG-IFNα) (n = 12, 24 weeks). Overall, the lower dose regimens achieved better antiviral responses at 12 weeks with improved tolerability, allowing the low dose regimens to be extended to 24 weeks. The primary endpoint of ≥2 log decline or <LLOQ of HDV RNA from baseline at end of treatment (EOT) was reached in 46% (6/13) and 89% (8/9) of patients receiving the all-oral regimen of lonafarnib 50 mg bid + ritonavir, and combination regimens of lonafarnib (25 or 50 mg bid) + ritonavir + PEG-IFNα, respectively. Other key findings included: (1) PEG-IFNα was found to be synergistic with lonafarnib (29% in oral, 63% in combo achieved 2 log decline in HDV RNA and ALT normalization); (2) patients presenting with lower (≤4 log) baseline viral loads had excellent responses to all oral lonafarnib 50 mg BID with RTV 100 mg BID, with 6/7 (86%) achieving HDV RNA levels below the LLOQ at 24 weeks EOT; (3) multiple patients experienced well-tolerated transient post-treatment ALT increases resulting in HDV RNA negativity and ALT normalization; (4) importantly, the regimens to advance into the phase 3 registration study were identified [49]. Indeed, the phase 3 *D-LIVR* study—the first and largest FDA-approved study to seek registration for HDV—is evaluating the all oral lonafarnib 50 mg BID with RTV 100 mg BID regimen alone, and in combination with PEG-IFNα, with a primary endpoint of HDV RNA ≥ 2 log decline and ALT normalization after 48 weeks of treatment. Secondary endpoints include improvement in histology. The study is fully enrolled and topline data is expected by the end of 2022 (clinicaltrials.gov # NCT03719313). Oral lonafarnib shows promise as a convenient treatment option in the future HDV treatment paradigm as a monotherapy and as an antiviral cocktail component in treatments for HDV.

### 3.4. Activating Innate Immunity—Peginterferon Lambda

Interferons (IFN) are a family of cytokines that possess various activities upon binding to their receptors, including immunoregulatory, antiviral, antiproliferative and more [50]. The antiviral activity of interferons made these cytokines the main weapon of defense against viral infection when no specific viral therapy was available. The most used interferons for antiviral therapy are type I interferons and indeed, IFNα was used extensively to treat many viral infections [51], and was, historically, the only agent, despite not being approved, used to treat HDV-infected patients. However, patient compliance is difficult due to the significant side effects associated with this therapy. These side effects include, but are not limited to, flu-like symptoms, depression and cytopenias, making it very difficult for patients to tolerate a full course of therapy [52]. A well-tolerated interferon, with comparable antiviral potency to PEG-IFNα, but with much better tolerability, is needed. As detailed next, this is the compelling case for peginterferon lambda (PEG-IFNλ).

The discovery of IFNλ, a type III IFN, added an exciting potential alternative to the arsenal of IFNs used to combat viral diseases [53]. While the post-receptor antiviral signaling is comparable between type I and type III IFNs (Figure 1d), the receptors for type III IFN are localized mostly on epithelial cells including in the liver and lungs [54] (Figure 1e). In contrast, the receptors for type I IFNs are present on almost every cell in the body, including immune cells (Figure 1e). This difference in the receptor tissue distribution accounts for type I IFN’s pleiotropic side effects and its potential to exacerbate a virus-induced cytokine storm [55]. Type III IFNλ is much better tolerated. This has been well documented in large randomized controlled studies comparing PEG-IFNα to PEG-IFNλ for the treatment of HCV and HBV [56,57,58], which showed better tolerability of PEG-IFNλ across the range of PEG-IFNα‘s classical side effects, with similar antiviral efficacy.

PEG-IFNλ was, therefore, an obvious and compelling candidate to evaluate for HDV therapy. The phase 2 *LIMT-1* (clinicaltrials.gov # NCT02765802) study enrolled 33 patients into an open-label randomized study with two doses of PEG-IFNλ, 120 micrograms (mcg) and 180 mcg, administered once weekly, subcutaneously for 48 weeks with a 24-week follow-up; 73% of patients completed the study demonstrating high compliance rate with the treatment regimen [59]. Historically, PEG-IFNα treatment achieves ~2.5 log decline in serum HDV RNA at the end of 48 weeks of treatment, with only 16% of patients achieving HDV RNA negativity 24 weeks after therapy [60]. The *LIMT-1* study showed a comparable decline in serum HDV RNA of 2.4 log at 180 mcg, and dose-proportional 1.4 log decline with 120 mcg [59]. Importantly, at the end of the 24-week follow-up, 36% of the patients treated with 180 mcg PEG-IFNλ achieved a durable virologic response (DVR), defined as HDV RNA levels below the limit of quantitation at 24 weeks post-treatment [59]. The *LIMT-1* study included per protocol dose reductions and drug discontinuations if a patient experienced a pre-defined lab abnormality. No clinical manifestations were observed with these lab abnormalities, and all lab abnormalities normalized with dose reduction and/or drug discontinuation. Moreover, even for patients who did not complete the study due to premature termination because of asymptomatic prespecified lab thresholds, 2/9 (22%) achieved a DVR in spite of receiving less than the intended amount of interferon lambda (O. Etzion, personal communication). While liver biopsies were not required as part of *LIMT-1* protocol, there were two patients who had liver biopsies performed pre- and 18 months post-treatment with PEG-IFNλ treatment. Dramatic regressions in fibrosis were observed, and this is the first report demonstrating fibrosis regression following finite duration therapy with PEG-IFNλ in patients with chronic HDV. These case studies suggest clinical benefit in the liver after 48 weeks of PEG-IFNλ therapy in the absence of HDV clearance [61].

These results clearly show that PEG-IFNλ can be a superior replacement for PEG-IFNα in future combination studies. Moreover, PEG-IFNλ is the only novel anti-HDV agent that prevents the HBsAg-independent cell-to-cell spread of HDV [62]. PEG-IFNλ is currently in the phase 3 *LIMT-2* study (clinicaltrials.gov # NCT05070364).

## 4. Combinations of Novel Therapies for HDV

Combination therapy has the benefit of reducing the risk of resistance development. Moreover, combination therapy can result in additive or even synergistic efficacy, enabling greater antiviral responses [63] and may allow lower doses to be used. As mentioned above, HDV patients with low baseline viral loads respond very well to all oral lonafarnib. For HDV patients with higher levels of virus, combination therapies can help maximize antiviral responses. As all novel therapies currently in clinical development for HDV work by different mechanisms of action, there are multiple potential combinations of novel anti-HDV therapies. The first of such combinations leveraged the successes of the phase 2 *LOWR-2* study—which demonstrated compelling antiviral synergy between lonafarnib and PEG-IFNα—and the Phase 2 *LIMT-1* study—which demonstrated PEG-IFNλ’s anti-HDV efficacy without the unwanted side effects of PEG-IFNα. In the Phase 2 *LIFT-1* study (clinicaltrials.gov # NCT03600714), conducted at the NIH, PEG-IFNλ 180 mcg once weekly was combined with lonafarnib 50 mg BID plus RTV 100 mg BID to treat 26 patients for 24 weeks with 24-week follow-up; 17/22 patients (77%) achieved the primary endpoint of >2 log decline in HDV RNA at EOT, and 11/22 patients (50%) were HDV RNA below the limit of quantitation or undetectable at EOT; 5/22 (23%) had a durable virologic response (DVR); 6/20 (30%) achieved the secondary endpoint of >2-point improvement in histologic activity index (HAI) at 24 weeks post therapy. Adverse events were mostly mild to moderate and included GI-related side effects [64]. The phase 2 *LIFT-2* study to be conducted at the NIH will explore a similar lonafarnib and PEG-IFNλ combination extended to 48 weeks of treatment.

## 5. Future Potential Combination Therapies

The expanding landscape of HDV therapy, with novel agents that target distinct stages in the HDV life cycle, will benefit HDV patients through combination therapy. While, to date, the first successful combination of novel agents is limited to Lonafarnib and PEG-IFNλ, these may be joined by inhibitors of HDV entry and HBsAg secretion. Side effects of these novel therapies are generally well tolerated and should not preclude such future combinations (although this will have to be experimentally determined). We are thus poised to embark on an exciting new era for HDV, the most severe form of human viral hepatitis.
